# Endovascular treatment in bilateral cavernous sinus dural arteriovenous fistulas: a systematic review and meta-analysis

**DOI:** 10.1038/s41598-023-31864-6

**Published:** 2023-08-01

**Authors:** Pang-Shuo Perng, Yu Chang, Yuan-Ting Sun, Hao-Kuang Wang, Yu-Shu Jiang, Jung-Shun Lee, Liang-Chao Wang, Chih-Yuan Huang

**Affiliations:** 1 Division of Neurosurgery, Department of Surgery, National Cheng Kung University Hospital, College of Medicine, National Cheng Kung University, No. 138, Sheng-Li Road, Tainan, 70428 Taiwan; 2 Department of Neurology, National Cheng Kung University Hospital, College of Medicine, National Cheng Kung University, Tainan, Taiwan; 3 Advanced Optoelectronic Technology Center, National Cheng Kung University, Tainan, Taiwan; 4 School of Medicine for International Students, I-Shou University, Kaohsiung, Taiwan; 5 Department of Neurosurgery, E-Da Hospital, I-Shou University, Kaohsiung, Taiwan; 6 Department of Surgery, National Cheng Kung University Hospital, College of Medicine, National Cheng Kung University, Tainan, Taiwan; 7 Department of Cell Biology and Anatomy, College of Medicine, National Cheng Kung University, Tainan, Taiwan; 8 Institute of Basic Medical Sciences, College of Medicine, National Cheng Kung University, Tainan, Taiwan

**Keywords:** Anatomy, Neurology, Oculomotor system

## Abstract

Few studies have discussed the disease nature and treatment outcomes for bilateral cavernous sinus dural arteriovenous fistula (CSDAVF). This study aimed to investigate the clinical features and treatment outcomes of bilateral CSDAVF. Embase, Medline, and Cochrane library were searched for studies that specified the outcomes of bilateral CSDAVF from inception to April 2022. The classification, clinical presentation, angiographic feature, surgical approach, and treatment outcomes were collected. Meta-analysis was performed using the random effects model. Eight studies reporting 97 patients were included. The clinical presentation was mainly orbital (n = 80), cavernous (n = 52) and cerebral (n = 5) symptoms. The most approached surgical route was inferior petrosal sinus (n = 80), followed by superior orbital vein (n = 10), and alternative approach (n = 7). Clinical symptoms of 88% of the patients (95% CI 80–93%, I^2^ = 0%) were cured, and 82% (95% CI 70–90%, I^2^ = 7%) had angiographic complete obliteration of fistulas during follow up. The overall complication rate was 18% (95% CI 11–27%, I^2^ = 0%). Therefore, endovascular treatment is an effective treatment for bilateral CSDAVF regarding clinical or angiographic outcomes. However, detailed evaluation of preoperative images and comprehensive surgical planning of the approach route are mandatory owing to complexity of the lesions.

## Introduction

Cavernous sinus dural arteriovenous fistula (CSDAVF) is the abnormal connection between arteries and veins within the cavernous sinus^1^. Most CSDAVF occurs unilaterally; however, bilateral CSDAVF have also been observed in some patients, including 14.2–26% of patients who suffer from CSDAVF^2,3^. The definition of bilateral CSDAVF is that the fistulas at each cavernous sinus have individual feeding arteries and venous drainage that can be visualized using highly selective digital subtraction angiography (DSA)^2^. Despite the possibility of spontaneous resolution^4^, patients with bilateral CSDAVF are prone to persistent neuro-ophthalmologic deficits and risk of intracranial hemorrhage, which prompt treatment^5^. Conventionally, these patients are treated with local compression, radiosurgery, and surgical ligation of the feeding arteries^6–8^. Endovascular treatment modalities have progressed since the millennium and are becoming the first treatment choice for CSDAVF^9^.

Unlike the comprehensive understanding of unilateral CSDAVF^10,11^, the clinical picture and related outcomes of endovascular treatment of bilateral CSDAVF are still under investigation. A greater hemodynamic impact is observed owing to more feeders and drainage veins^12^, and more complicated anatomies with difficult treatment strategies separate bilateral CSDAVF from unilateral CSDAVF^3^. Owing to the aforementioned reasons, we performed a systematic review of pertinent studies with the aim of illustrating the current classification, clinical symptoms and signs, approach techniques, clinical-angiographic outcomes, and complication rate of bilateral CSDAVF treated in an endovascular fashion.

## Materials and method

### Literature search and inclusion and exclusion criteria

This systematic review was conducted according to the Preferred Reporting Items for Systematic Reviews and Meta-Analyses statement (PRISMA). The Cochrane Library, Embase, and Medline electronic databases were searched from inception to April 4, 2022. The search terms used were “cavernous sinus,” “carotid-cavernous,” “carotid cavernous,” “dural arteriovenous fistula,” and “carotid-cavernous fistula.” “Patient, Intervention, Comparison, and Outcome” were the outcome of endovascular therapy for bilateral CSDAVF. The study protocol was registered in PROSPERO (CRD42022338792). The detailed search strategy and PRISMA checklist are presented in the Supplemental Table 1 and 2.

**Table 1:** Characteristics of included studies.

Study	Country	Study type	Patient number	Follow up	Female (%)	Age, mean (SD)	Approach route	Complication rate	Clinical Cured (%)	Angiographic Cured (%)
Klisch 2003	Germany	Retrospective	3	Median 7 months	100	68.7 (5.0)	Unilateral IPS: 1, SOV: 1, combine transarterial and transvenous: 1	33.3% transient amnesia and apraxia, SAH	66.6	66.6
Wahloo 2007	USA	Retrospective	5	6 to 36 months	60	67.8 (20)	None specified IPS: 3, SOV: 2	20% transient CN VI palsy, 20% IPS injury, 20% nBCA leakage	100	100
LV 2008	China	Retrospective	3	Median 12 months	66.7	51 (11.4)	Bilateral IPS : 2, unilateral IPS: 1	66.6% transient CN VI palsy	100	NR
Hassan 2015	Egypt	Retrospective	3	6 to 60 months	66.7	55 (5)	Unilateral IPS: 2, SOV: 1	0%	100	100
Rhim 2017	Korea	Retrospective	17	Mean 33.7 month	76.5	64.9 (NR)	Bilateral IPS : 9, unilateral IPS: 7, Facial vein: 1	23.5% transient CN palsy, 17.6% permanent CN palsy	82.4	100
Fay 2019	Taiwan	Retrospective	20	Post operation 1 year and every 6 month	90	64.3 (11.7)	Bilateral IPS : 13, unilateral IPS: 4, facial vein: 3	15% transient CN palsy	95	97.5
Nossek 2020	USA	Retrospective	3	Median 9 months	33.3	65.7 (12.7)	Unilateral IPS: 3	0%	100	100
Churojana 2021	Thialand	Retrospective	43	Median 23.5 months	83.7	61.8 (11.0)	Bilateral IPS : 5, unilateral IPS: 30, combine transarterial and transvenous: 2, SOV: 2, transarterial: 1, manual compression: 3	6.9% IPS injury, 4.7% transient CN palsy	90.7	74.4

CN: cranial nerve, IPS: inferior petrosal sinus, NR: not reported, SOV: superior ophthalmic vein, SAH: subarachnoid hemorrhage.

**Table 2:** Demographics of the included patient, presented with raw and pooled proportions.

		Patients(n/N)	Raw proportions (95% CI)	Pooled proportions (95% CI)	I^2^ (%)
Sex	Male	19/97	20 (11–45)	21% (14–31)	0
	Female	78/97	80 (55–89)	79% (69–86)	0
Barrow classification	C	3/34	9 (0–17)	12% (5–28)	0
	D	31/34	91 (83–100)	88% (72–95)	0
Cognard classification	I	3/26	12 (0–18)	16% (6–34)	0
	IIa	10/26	38 (0–61)	29% (8–66)	43
	IIa+b	8/26	31 (0–92)	36% (15–64)	24
	III	5/26	19 (0–100)	32% (6–78)	57
Satomi classification	1	16/43	37 (N/A)		
	2	13/43	30 (N/A)		
	3	14/43	33 (N/A)		
Clinical presentation	Oribital	80/97	82 (78–100)	80% (71–87)	0
	Cavernous	52/97	54 (52–91)	65% (43–82)	68
	Cerebral	5/97	5 (0–12)	14% (8–23)	0
Bilateral symptoms		41/89	46 (34–84)	45% (35–56)	0
IPS occlusion	Unilateral	16/80	27 (0–74)	22% (6–57)	80
	Bilateral	11/80	14 (0–39)	15% (7–28)	31
Approach route	IPS	80/97	85 (58–97)	80% (70–87)	0
	Superior ophthalmic vein	10/97	10 (3–30)	15% (7–27)	9
	Alternative method	7/97	4 (0–16)	12% (7–21)	0
Embolization agents	Coils	58/94	62 (10–85)	51% (16–85)	76
	Coils +/- Onyx or nBCA	30/94	32 (7–76)	37% (10–75)	75
	Onyx	1/94	1 (0–14)	7% (3–18)	0
	nBCA	5/94	5 (0–18)	11% (5–22)	0

CI: confidence interval, IPS: inferior petrosal sinus , nBCA: N-butyl cyanoacrylate.

Articles were included according to the following criteria: 1. studies that reported at least three patients diagnosed with bilateral CSDAVF, 2. results obtained from these patients must have been specified and separated from the unilateral CSDAVF, including clinical or image outcomes, and 3. the articles must have been written in English. Articles were excluded when one of the following was noted: 1. pediatric outcomes; 2. non-human studies; and 3. article types such as case reports, editorials, letters to the editor, review articles, and conference abstracts. When institutions had duplicate studies with different numbers of patients or increased follow-up lengths, only the most complete reports on bilateral CSDAVF were included. Two investigators (P.S.P. and Y.C.) independently performed the search to identify relevant studies for inclusion, with a third investigator (C.Y.H.) resolving discrepancies throughout the database search phase.

### Data extraction and quality assessment

Two investigators (P.S.P. and Y.C.) independently extracted the following data from the included studies: publication year, country where the study was conducted, first author's last name, study population, clinical presentation, classification of the bilateral CSDAVF, endovascular treatment method, surgical approach, clinical and angiographic outcomes, and complication rate. The orbital symptoms were the symptoms related to orbital area, including blurred vision, ocular pain, chemosis, proptosis, and hemorrhage in the ocular structure. The cavernous symptoms were related to the involvement of the cranial nerve, which included diplopia, ptosis, anisocoria, and ophthalmoplegia. The cerebral symptoms included focal neurological signs including motor or sensory deficits, seizure, and intracranial hemorrhage. The classification of the symptoms was based on the venous drainage pattern and symptoms caused by elevated venous output. Two investigators (P.S.P. and Y.C.) independently utilized the Cochrane risk of bias in non-randomized intervention studies to critically appraise the included literature (Supplemental Fig. 1). Discordances were resolved by consulting the senior author (C.Y.H.).

**Figure 1:**
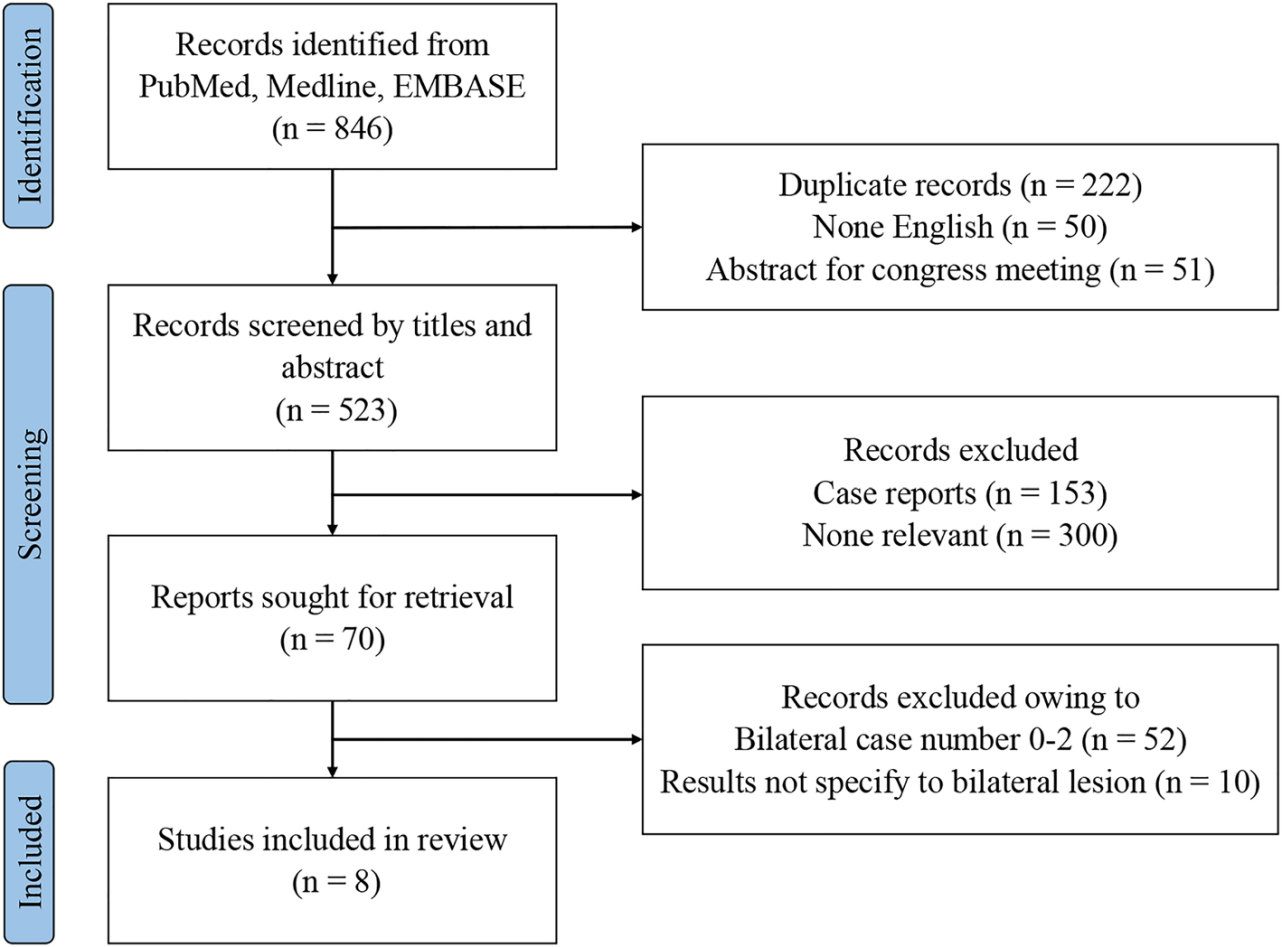
The searching flowchart.

### Statistical analysis

The article results were pooled with a proportional meta-analysis using the random-effect model. Statistical heterogeneity was measured using the Higgins’ index (I^2^), with I^2^ > 50% indicating a significantly high heterogeneity. The *p* values were two-sided, and a statistically significant difference was defined as *p* < 0.05. Potential publication bias was not tested, owing to the nature and study number of the meta-analysis. The analysis was performed using R software, version 4.1.3. (United States).

## Results

### Study selection

Using the search terms, 846 articles were retrieved from the database. After excluding duplicate studies, conference abstracts, and articles not in English, the remaining 445 references were screened using titles and abstracts. Seventy relevant articles were retrieved for a full-length article review, and 10 studies were excluded because they did not specify the results of the bilateral CSDAVF, and 52 studies owing to less case numbers (Supplemental Tables 3 and 4). The remaining eight articles were included in the review (Table 1)^2,3,13–18^. The process is summarized in Fig. 1.

### Demographic characteristics and clinical symptoms

Data of 97 patients were included in this review (Table 2). Among them, 78 were women (80%) and 19 were men (20%). The mean patient age was 63 years. The patients were classified according to Barrow in three studies, Cognard in three studies, and Satomi in one study. Most of the patients were in Barrow (Type D; 91%) and Cognard (at least Type IIa; 88%). In this review, bilateral symptoms presented in 45% (95% confidence interval [CI], 35–56%, I^2^ = 0%) of the patients, excluding eight patients who did not record the side of the symptoms. The most common symptoms observed in the reviewed studies after pooling were orbital symptoms in 80% (95% CI 71–87%, I^2^ = 0%) of the patients, followed by cavernous and cerebral symptoms, in 65% (95% CI 43–82%, I^2^ = 68%) and 14% (95% CI 8–23%, I^2^ = 0%) of the patients, respectively. In 77 patients with detailed preoperative symptoms, 43 (56%) had chemosis, 17 (22%) had blurred vision, 14 (18%) had cranial nerve palsy, 14 (18%) had proptosis, 10 (13%) had ophthalmoplegia, 8 (10%) had tinnitus, 3 (4%) had headache, and one (1%) patient had motor deficits.

### Surgical approach and embolization agents

All the reviewed patients underwent endovascular treatment via the transvenous approach, except for four patients who received an additional transarterial approach and three patients with additional manual compression. The endovascular procedures were performed via unilateral inferior petrosal sinus (IPS) catheterization in 48 (53%) patients, bilateral IPS catheterization in 29 (32%) patients, an approach with the side of IPS not specified in three (3%) patients, and the superior ophthalmic vein (SOV) approach in 10 (11%) patients. Unilateral IPS and bilateral IPS occlusion rates were reported in three studies, with a pooled incidence of 22% (95% CI 6–57%, I^2^ = 80%) and 15% (95% CI 7–28%, I^2^ = 31%), respectively^2,3,17^. Embolization agents were mainly coils in 58 (62%) patients and coils combined with N-butyl cyanoacrylate (nBCA) or onyx in 30 (32%) patients. Three other (3%) patients received nBCA only, and one (1%) patient received onyx only. One study reported the mean coil length and onyx amount, which were 150 ± 88 cm for each lesion and 1.6 ± 0.4 mL, respectively^3^.

### Outcomes and complication

**Figure 2:**
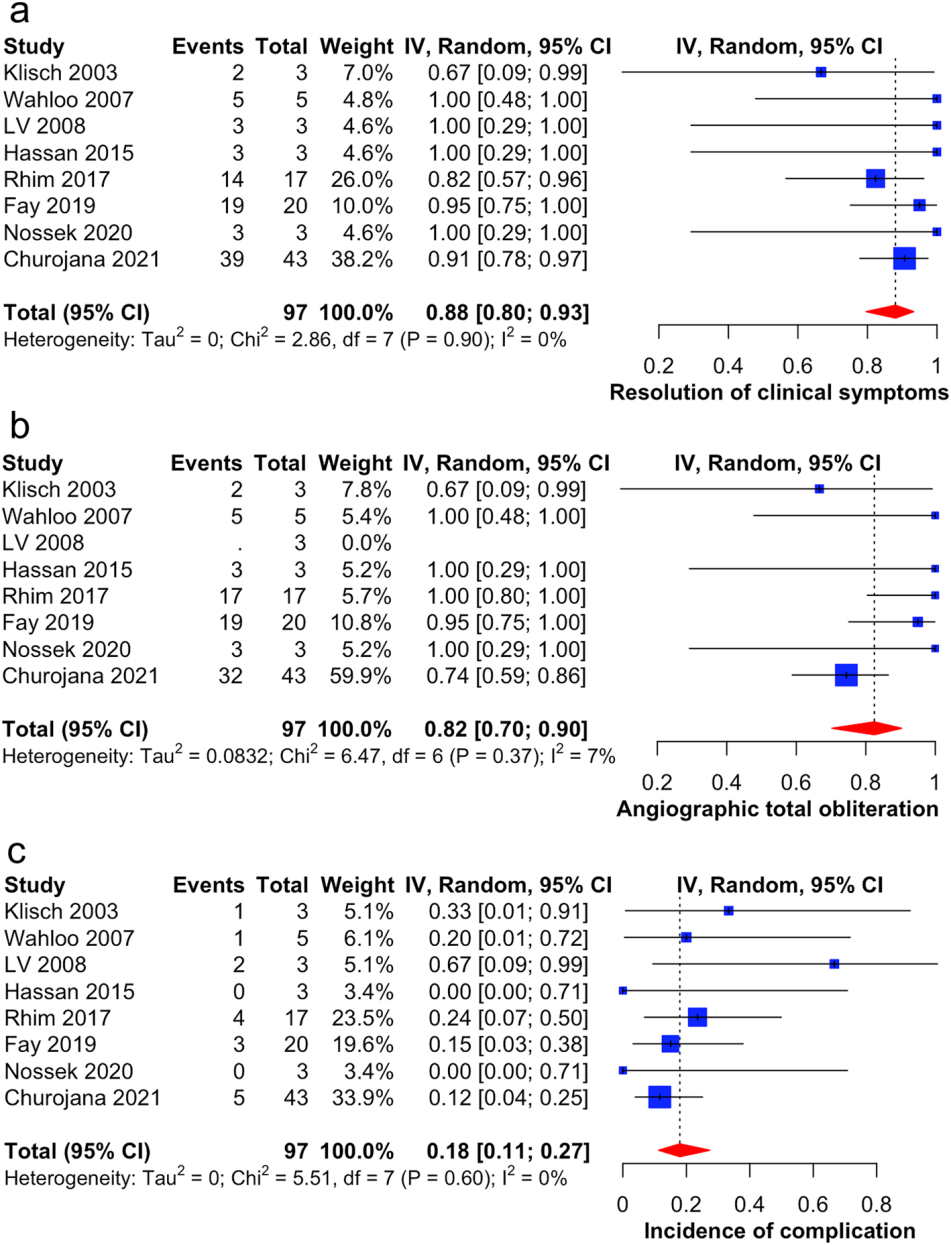
The outcomes of bilateral CSDAVF after endovascular treatment using random-effect meta-analysis. The clinical resolution rate and angiographic obliteration rate were presented in (**a**) and (**b**), respectively; section (**c**) illustrates the complication rate after treatment.

After a pooled analysis, clinical symptoms were cured in 88% of the patients (95% CI 80–93%, I^2^ = 0%) and complete angiographic obliteration of fistula during follow-up was noted in 82% of the patients (95% CI 70–90%, I^2^ = 7%) (Fig. 2A,B). The overall complication rate was 18% (95% CI 11–27%, I^2^ = 0%) (Fig. 2C). The most common complication of bilateral CSDAVF for endovascular treatment was cranial nerve palsy, with transient type in 12 patients (13%) and permanent type in four (4%) patients. Other complications also occurred, including IPS injury due to wire protruding through the vessel wall in four (4%) patients, hemorrhage in one (1%) patient, and nBCA leakage in one (1%) patient.

## Discussion

Bilateral CSDAVF is less studied than unilateral CSDAVF. To the best of our knowledge, this review is the first to emphasize bilateral lesions and analyze the outcomes. This systematic review included eight studies (97 patients). We found that an embolization via transvenous approach could reach an 88% (95% CI 80–93%, I^2^ = 0%) clinical cure rate and 82% (95% CI 70–90%, I^2^ = 7%) angiographic obliteration rate, which was comparable to those in the general population of CSDAVF^9,19,20^.

### Classification

Historically, researchers have classified CSDAVF according to the Barrow^21^, Cognard^22^, or Satomi^23^ classifications, with Cognard incorporating venous drainage as a risk factor and Satomi focusing on the outcomes. Although these two classifications were generated from general DAVF, they were specific to CSDAVF. More recently, Su et al.^24^ and Thomas et al.^25^ announced newer classifications in an attempt to summarize the complexity of the angiographic nature and clinical presentation of CSDAVF, which has been verified in recent studies^26,27^. However, none of these classifications were designed exclusively for bilateral CSDAVF. Wenderoth then reported the modified classification based on Cognard classification, adding a specific “c” classification for the bilateral group^28^. In addition, he specified the patency of each IPS for treatment planning. In this review, multiple classification methods were used, with five studies using Barrow classification, four studies using Cognard classification, and only one study reporting patients with the Satomi classification system. The heterogeneity was high between the studies; therefore, large-sample studies are warranted in the future to substantiate the associations of the classification with the nature and outcomes of bilateral CSDAVF.

### Clinical presentation

In the current study, orbital and cavernous symptoms were significantly more common than cerebral symptoms, with low heterogeneity (I^2^ = 0) in orbital symptoms and high heterogeneity (I^2^ = 68) in cavernous symptoms (Table 2). The largest cohort in our review^2^ had a lower cavernous symptom rate (23%) than others. Previous studies have shown the relationship between fistula drainage and clinical symptoms and concluded that anterior drainage may cause more orbital symptoms, while posterior drainage may cause more neurological symptoms^8^. A higher orbital symptom rate seemed to indicate a more indolent disease course. However, in a recent study, cortical venous reflux, which is strongly associated with intra-cerebral hemorrhage before treatment, mostly presented with chemosis or orbital pain^29^. Therefore, a comprehensive study including magnetic resonance imaging, computed tomography angiography, and DSA is warranted for patients with orbital symptoms to determine if pial venous reflux exists. In addition, interestingly, bilateral presentation was only observed in 46% of patients with bilateral CSDAVF in our review. Fay et al. attributed this to the direction of fistula flow^3^. Taken together, patients with suspicious symptoms and signs should be transferred to an experienced physician for full evaluation and sophisticated treatment plans.

### Surgical approach and nuance in bilateral lesions

IPS is usually the first choice for transvenous endovascular surgery owing to its simplicity, effectiveness, and the shortest connection with the cavernous sinus from the jugular bulb. In our review, the most common route was the IPS, with low heterogeneity. If the IPS route is chosen, a unilateral or bilateral approach can be applied to bilateral CSDAVF. However, in our review, unilateral or bilateral IPS occlusion rates were 22% (95% CI 6–57%, I^2^ = 80%) and 15% (95% CI 7–28%, I^2^ = 31%), respectively, similar to previously published data regarding CSDAVF^29,30^. The high variability could be caused by the limited number of studies and patient numbers. The IPS route becomes more important for bilateral CSDAVF because it would be difficult to completely obliterate lesions on each side via a single alternative route. Therefore, embolization via an occluded IPS has become challenging, but somehow an inevitable procedure. Multiple methods have been used to deal with occluded IPS, including “Pocket-Flash method,”^31^ “Frontier-Wire Probing technique,”^32^ and “microguidewire looping technique”^33^. However, some have opposed the breaching of the occluded IPS technique and considered it a dangerous maneuver^34^. In the current review, the IPS injury rate was 4% for bilateral CSDAVF.

**Figure 3:**
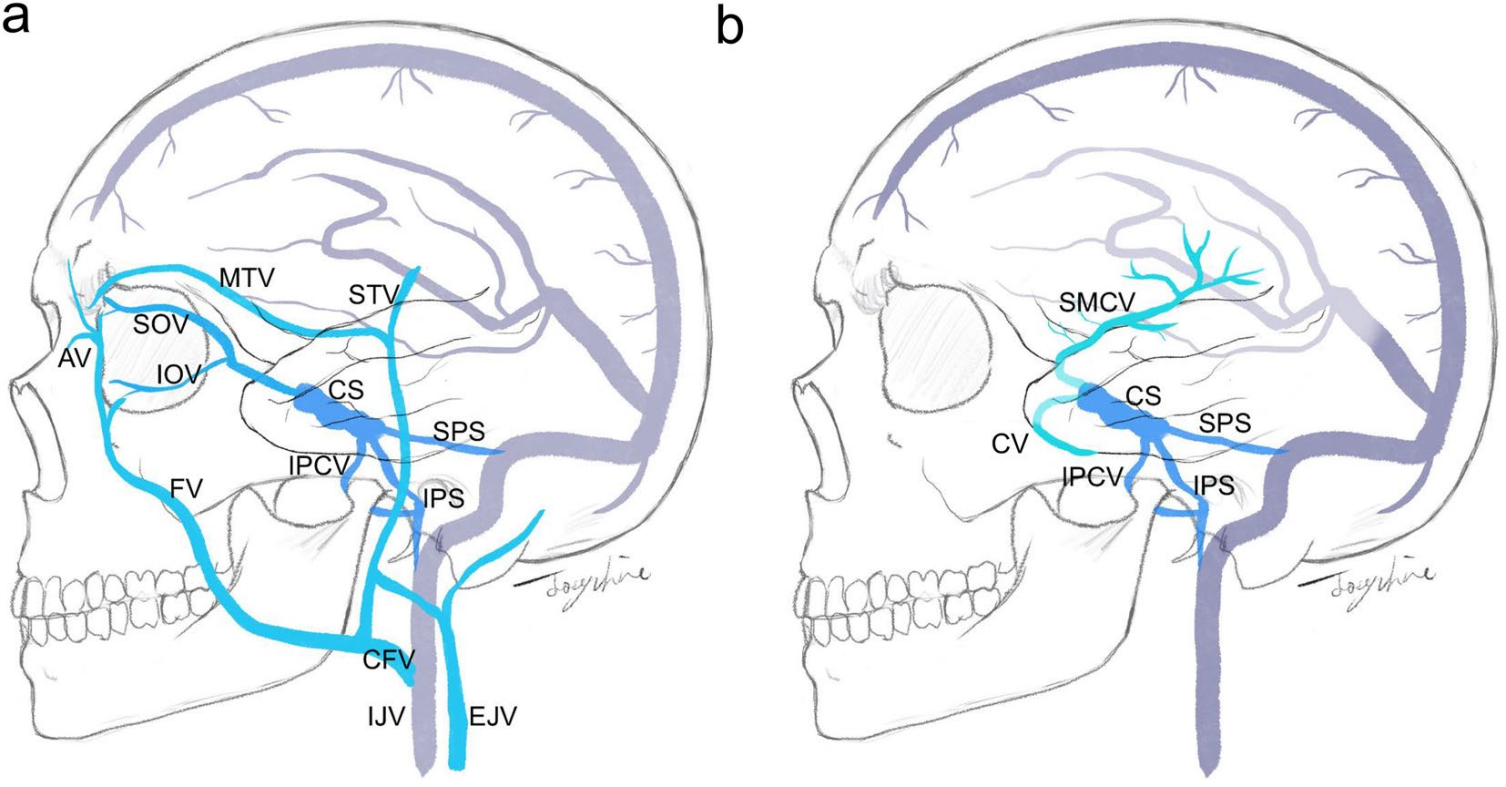
Different approach routes for transvenous cavernous sinus embolization from the superficial veins (**a**) and intracranial veins (**b**). The traditional route is IPS^35,36^ and SOV, including endovascular^34,38–41^, direct puncture^32,42–44^ and surgical cutdown^45,46^ for SOV approach. Other alternative routes include SPS^37^, SMCV^47,48^, IPCV^49,50^, CV^51–54^, in which some routes have to be approached after surgical exposure. AV: angular vein, CFV: common facial vein, CS: cavernous sinus, CV: cortical veins, EJV: external jugular vein, FV: facial vein, IJV: internal jugular vein, IOV: inferior ophthalmic vein, IPCV: inferior petroclival vein, IPS: inferior petrosal sinus, MTV: middle temporal vein, SOV: superior ophthalmic vein, SPS: superior petrosal sinus, STV: superficial temporal vein, SMCV: superficial middle cerebral vein.

Several other approach routes for CSDAVF have been reported and are summarized in Fig. 3. The SOV route has been previously reported to have a satisfactory embolization rate^38^. Direct puncture or surgical cutdown has been utilized to approach SOV and avoid the difficulty in navigating the catheters and the possibility of vessel wall injury during the procedure^34,41,44^. Possible complications include periorbital structural damage and hematoma^55,56^. However, in bilateral CSDAVF, if a unilateral approach is chosen, it would be more difficult to pass the cavernous sinus connection due to poor catheter support. On the other hand, bilateral SOV routes increase post-operative suffering and worsen cosmetic results^57^. Therefore, for bilateral CSDAVF, a unilateral or bilateral approach through the traditional IPS, facial vein, or SOV approach through a direct puncture or surgical cutdown are all reasonable choices, and detailed treatment plans should be made before the surgery and adjusted during the surgery.

Another issue for bilateral CSDAVF is whether a single-session or staged operation should be performed. Some previous studies have advocated staged operation for unilateral and bilateral CSDAVF^58,59^. The reasons were to reduce the coil amount, which has been proven to be associated with postoperative cranial nerve VI palsy^60,61^ owing to the anatomical features of this nerve^62–64^. In addition, the hemodynamic change between surgeries may also have the possibility of reducing the coils needed for second-stage surgery^3^. However, staged surgery still has some obstacles. Firstly, navigating the microcatheter into the venous pouch or through the connection of the cavernous sinus with the resistance of previous coils and onyx can be challenging, since “Turn-Back Embolization Technique” is usually applied^65^. Second, the timing of surgery can be ambiguous. Clinical embolization outcomes or paradoxical cranial nerve VI palsy are difficult to evaluate in this situation^66–68^. Single-stage surgeries have the advantage of avoiding these difficulties. Although no studies have compared the efficacy of single or multi-stage surgery for bilateral CSDAVF, a careful assessment of preoperative images to ensure that all the venous pouches and fistulas were targeted is of paramount importance as the opacity of the mass of coils that may hide a residual flow could be especially challenging in bilateral CSDAVF than in unilateral lesions during the surgery^69–71^.

### Complication rate

One of the included studies^15^ with only three patients had a higher complication rate (66.6%), reporting two transient cranial nerve palsy patients. The remaining studies in current review reported low complication rates. The most common complication was cranial nerve VI palsy, with 13% of the patients recovering spontaneously and 4% of the patients developing permanent nerve deficits. A previous meta-analysis of general CSDAVF group had a complication rate of 7.75% (95% CI 3.82–12.7%) with minimal permanent deficits (0.15%)^10^. In addition to the cranial nerve palsy and IPS injury mentioned above, leakage of embolization agents was also a possible consequence. Onyx or nBCA, which refluxes back into the feeding arteries, can result in non-target embolization and have catastrophic complications. This is especially important for bilateral lesions, since adjuvant onyx or nBCA is frequently used for complete embolization. In this study, none of the previously reported serious complications, such as brainstem infarction, brainstem hemorrhage, and intra-cerebral hemorrhage, were speculated to be related to the advancement of the techniques and were well aware of the anatomy of the related structures^67,72,73^. However, Wakhloo et al. reported a case of nBCA leakage without severe stroke or hemorrhagic episode^14^. Finally, in complications related to uncontrolled bleeding or strategies for endovascular bailout, surgery could always be considered to obliterate the fistula and achieve hemostasis^74,75^.

### Limitations

One of the main limitations of this systematic review was the retrospective design of the majority of the included studies, which was a potential source of bias due to confounding factors. In addition, with intension to reveal the whole picture of the disease, we included several studies with small case numbers. Therefore, the results had to be interpreted carefully. Second, the classification for CSDAVF was not uniform between the studies. Third, the definition of bilateral CSDAVF has not been clarified in previous studies. Fourth, a majority of study was excluded as they failed to report the outcomes specifically for bilateral lesions. This can lead to bias during data analysis. Fifth, none of the studies reported the intraocular pressure measurement before or after the treatment.

## Conclusion

Management of bilateral CSDAVF remains challenging. The patient can present with unilateral symptoms, which pose difficulties in disease diagnosis. The endovascular treatment strategies for bilateral CSDAVF should be tailored according to the patency of the IPS, accessibility of the SOV or other routes, and if staging operation is needed. According to meta-analysis of modest quality of data, weak suggestions can be made that a transvenous embolization is a feasible treatment method for bilateral CSDAVF.

## Supplementary Information

Supplementary Information.

## Data Availability

The data that support the findings of this study are available on request from the corresponding author.
