# Supplementary material for: Endovascular treatment in bilateral cavernous sinus dural arteriovenous fistulas: a systematic review and meta-analysis
Source: Sci Rep. 2023 Aug 1;13:7108. doi: 10.1038/s41598-023-31864-6 (PMC10394050; doi:10.1038/s41598-023-31864-6)

**Supplemental Table 1.**

| **Database** | **#** | **Search Description** | **Numbers of results** |
| --- | --- | --- | --- |
| **Embase** | 1 | (cavernous sinus or carotid-cavernous or carotid cavernous or carotico-cavernous):ti,ab,kw,de | 16712 |
|  | 2 | 'cavernous sinus'/exp | 8190 |
|  | 3 | dural arteriovenous fistula | 4809 |
|  | 4 | 'dural arteriovenous fistula'/exp | 2681 |
|  | 5 | (endovascular or transarterial or transvenous or intravascular):ti,ab,kw,de | 214075 |
|  | 6 | 'endovascular surgery'/exp | 45490 |
|  | 7 | (#1 OR #2 ) AND (#3 OR #4) AND (#5 OR #6) and [embase]/lim | 509 |
| **Medline** | 1 | (cavernous sinus or carotid-cavernous or carotid cavernous or carotico-cavernous).mp | 9843 |
|  | 2 | exp "Cavernous Sinus"/ | 4667 |
|  | 3 | dural arteriovenous fistula.mp | 2121 |
|  | 4 | exp "Central Nervous System Vascular Malformations"/ | 13664 |
|  | 5 | (endovascular or transarterial or transvenous or intravascular).mp | 134104 |
|  | 6 | exp "Endovascular Procedures"/ | 137555 |
|  | 7 | (1 OR 2) AND (3 OR 4) AND (5 OR 6) | 337 |
| **Cochrane** | 1 | cavernous sinus or carotid-cavernous or carotid cavernous or carotico-cavernous | 62 |
|  | 2 | MeSH descriptor: [Cavernous sinus] explode all trees | 9 |
|  | 3 | Dural arteriovenous fistula | 20 |
|  | 5 | MeSH descriptor; [Central nerve system vascular malformations] explode all trees | 81 |
|  | 6 | Endovascular or transvenous or intravascular | 10429 |
|  | 7 | MeSH descriptor; [Endovascular procedures] explode all trees | 9218 |
|  | 8 | (1 OR 2) AND (4 OR 5) AND (6 OR 7) | 0 |

**Supplemental Table 2.**

| **Section and Topic** | **Item #** | **Checklist item** | **Location where item is reported** |
| --- | --- | --- | --- |
| **TITLE** | | |  |
| Title | 1 | Identify the report as a systematic review. | Page 1 |
| **ABSTRACT** | | |  |
| Abstract | 2 | See the PRISMA 2020 for Abstracts checklist. | Page 2 |
| **INTRODUCTION** | | |  |
| Rationale | 3 | Describe the rationale for the review in the context of existing knowledge. | Page 2, 3 |
| Objectives | 4 | Provide an explicit statement of the objective(s) or question(s) the review addresses. | Page 2, 3 |
| **METHODS** | | |  |
| Eligibility criteria | 5 | Specify the inclusion and exclusion criteria for the review and how studies were grouped for the syntheses. | Page 3 |
| Information sources | 6 | Specify all databases, registers, websites, organisations, reference lists and other sources searched or consulted to identify studies. Specify the date when each source was last searched or consulted. | Page 3 |
| Search strategy | 7 | Present the full search strategies for all databases, registers and websites, including any filters and limits used. | Supplement Table 1 |
| Selection process | 8 | Specify the methods used to decide whether a study met the inclusion criteria of the review, including how many reviewers screened each record and each report retrieved, whether they worked independently, and if applicable, details of automation tools used in the process. | Figure 1 |
| Data collection process | 9 | Specify the methods used to collect data from reports, including how many reviewers collected data from each report, whether they worked independently, any processes for obtaining or confirming data from study investigators, and if applicable, details of automation tools used in the process. | Page 3, 4 |
| Data items | 10a | List and define all outcomes for which data were sought. Specify whether all results that were compatible with each outcome domain in each study were sought (e.g. for all measures, time points, analyses), and if not, the methods used to decide which results to collect. | Page 4 |
|  | 10b | List and define all other variables for which data were sought (e.g. participant and intervention characteristics, funding sources). Describe any assumptions made about any missing or unclear information. | Page 4 |
| Study risk of bias assessment | 11 | Specify the methods used to assess risk of bias in the included studies, including details of the tool(s) used, how many reviewers assessed each study and whether they worked independently, and if applicable, details of automation tools used in the process. | Supplemental Figure 1 |
| Effect measures | 12 | Specify for each outcome the effect measure(s) (e.g. risk ratio, mean difference) used in the synthesis or presentation of results. | Page 4 |
| Synthesis methods | 13a | Describe the processes used to decide which studies were eligible for each synthesis (e.g. tabulating the study intervention characteristics and comparing against the planned groups for each synthesis (item #5)). | Page 4 |
|  | 13b | Describe any methods required to prepare the data for presentation or synthesis, such as handling of missing summary statistics, or data conversions. | Page 4 |
|  | 13c | Describe any methods used to tabulate or visually display results of individual studies and syntheses. | Page 4 |
|  | 13d | Describe any methods used to synthesize results and provide a rationale for the choice(s). If meta-analysis was performed, describe the model(s), method(s) to identify the presence and extent of statistical heterogeneity, and software package(s) used. | Page 4 |
|  | 13e | Describe any methods used to explore possible causes of heterogeneity among study results (e.g. subgroup analysis, meta-regression). | Page 4 |
|  | 13f | Describe any sensitivity analyses conducted to assess robustness of the synthesized results. | Page 4 |
| Reporting bias assessment | 14 | Describe any methods used to assess risk of bias due to missing results in a synthesis (arising from reporting biases). | N/A |
| Certainty assessment | 15 | Describe any methods used to assess certainty (or confidence) in the body of evidence for an outcome. | N/A |
| **RESULTS** | | |  |
| Study selection | 16a | Describe the results of the search and selection process, from the number of records identified in the search to the number of studies included in the review, ideally using a flow diagram. | Figure 1 |
|  | 16b | Cite studies that might appear to meet the inclusion criteria, but which were excluded, and explain why they were excluded. | Supplement Table 3 and 4 |
| Study characteristics | 17 | Cite each included study and present its characteristics. | Table 1 |
| Risk of bias in studies | 18 | Present assessments of risk of bias for each included study. | Supplement Figure 1 |
| Results of individual studies | 19 | For all outcomes, present, for each study: (a) summary statistics for each group (where appropriate) and (b) an effect estimate and its precision (e.g. confidence/credible interval), ideally using structured tables or plots. | Table 2 |
| Results of syntheses | 20a | For each synthesis, briefly summarise the characteristics and risk of bias among contributing studies. | Figure 2 |
|  | 20b | Present results of all statistical syntheses conducted. If meta-analysis was done, present for each the summary estimate and its precision (e.g. confidence/credible interval) and measures of statistical heterogeneity. If comparing groups, describe the direction of the effect. | Figure 2 |
|  | 20c | Present results of all investigations of possible causes of heterogeneity among study results. | Figure 2 |
|  | 20d | Present results of all sensitivity analyses conducted to assess the robustness of the synthesized results. | N/A |
| Reporting biases | 21 | Present assessments of risk of bias due to missing results (arising from reporting biases) for each synthesis assessed. | Supplement Figure 1 |
| Certainty of evidence | 22 | Present assessments of certainty (or confidence) in the body of evidence for each outcome assessed. | N/A |
| **DISCUSSION** | | |  |
| Discussion | 23a | Provide a general interpretation of the results in the context of other evidence. | Page 5 |
|  | 23b | Discuss any limitations of the evidence included in the review. | Page 8 |
|  | 23c | Discuss any limitations of the review processes used. | Page 5-8 |
|  | 23d | Discuss implications of the results for practice, policy, and future research. | Page 5-8 |
| **OTHER INFORMATION** | | |  |
| Registration and protocol | 24a | Provide registration information for the review, including register name and registration number, or state that the review was not registered. | Page 3 |
|  | 24b | Indicate where the review protocol can be accessed, or state that a protocol was not prepared. | Page 3 |
|  | 24c | Describe and explain any amendments to information provided at registration or in the protocol. | Page 3 |
| Support | 25 | Describe sources of financial or non-financial support for the review, and the role of the funders or sponsors in the review. | N/A |
| Competing interests | 26 | Declare any competing interests of review authors. | N/A |
| Availability of data, code and other materials | 27 | Report which of the following are publicly available and where they can be found: template data collection forms; data extracted from included studies; data used for all analyses; analytic code; any other materials used in the review. | N/A |

**Supplemental Table 3 Studies not specifying the bilateral results**

| Year | Author | Title |  |
| --- | --- | --- | --- |
| 2017 | Afonso et al. | Transvenous embolization of dural carotid cavernous fistulas: the role of liquid embolic agents in association with coils on patient outcomes | |
| 2018 | Alexander et al. | Long-Term Outcomes of Endovascular Treatment of Indirect Carotid Cavernous Fistulae: Superior Efficacy, Safety, and Durability of Transvenous Coiling Over Other Techniques | |
| 2020 | Ertl et al. | Patient reported long-term outcome after endovascular therapy of indirect dural carotid cavernous fistula | |
| 2019 | Holland et al. | Endovascular treatment of carotid–cavernous sinus fistulas: ophthalmic and visual outcomes | |
| 2018 | Jia et al. | Cannulation of Occluded Inferior Petrosal Sinuses for the Transvenous Embolization of Cavernous Sinus Dural Arteriovenous Fistulas: Usefulness of a Frontier-Wire Probing Technique | |
| 2017 | Nishimuta et al. | Long-term outcome after endovascular treatment of cavernous sinus dural arteriovenous fistula and a literature review | |
| 2016 | Pashapour et al. | Long-Term Endovascular Treatment Outcome of 46 Patients with Cavernous Sinus Dural Arteriovenous Fistulas Presenting with Ophthalmic Symptoms | |
| 2010 | Yoshida et al. | Transvenous Embolization of Dural Carotid Cavernous Fistulas: A Series of 44 Consecutive Patients | |
| 2007 | Yu et al. | TRANSVENOUS EMBOLIZATION OF DURAL CAROTID-CAVERNOUS FISTULAE WITH TRANSFACIAL CATHETERIZATION THROUGH THE SUPERIOR OPHTHALMIC VEIN | |
| 2011 | Kurata et al. | Dural Arteriovenous Fistulas in the Cavernous Sinus: Clinical Research and Treatment | |

**Supplemental Table 4 Studies with less case numbers**

| Year | Author | Title |
| --- | --- | --- |
| 2021 | Ide et al. | Selective transvenous embolization combined with balloon angioplasty of the occluded inferior petrosal sinus for the treatment of cavernous sinus dural arteriovenous fistulas |
| 2021 | Zhang et al. | Embolization of Cavernous Sinus Dural Arteriovenous Fistula (CSDAVF) via transvenous approaches: Practice, experience summary and literature review |
| 2021 | Guedon et al. | Results of transvenous embolization of intracranial dural arteriovenous fistula: a consecutive series of 136 patients with 142 fistulas |
| 2021 | Fujita et al. | Impact of transvenous embolization via superior ophthalmic vein on reducing the total number of coils used for patients with cavernous sinus dural arteriovenous fistula |
| 2019 | Xu et al. | Treatment of cavernous sinus dural arteriovenous fistula using different surgical approaches: Analysis of 32 consecutive cases |
| 2019 | Leone et al. | Carotid Cavernous Fistulas and Dural Arteriovenous Fistulas of the Cavernous Sinus: Validation of a New Classification According to Venous Drainage |
| 2019 | Choi et al. | Making Microguidewire Loop Facilitates Navigation Through Tortuous or Abruptly Angulated Head and Neck Veins to Access Cavernous Sinus Dural Arteriovenous Fistulas |
| 2018 | Kohta et al. | Novel Segmentation of Placed Coils in the Treatment of Cavernous Sinus Dural Arteriovenous Fistulas Provides a Reliable Predictor of the Long-Term Outcome in Abducens Nerve Palsy |
| 2018 | Trivelato et al. | Transorbital Cavernous Sinus Direct Puncture Alternative to treat dural arteriovenous fistula |
| 2016 | Wenderoth et al. | Novel approaches to access and treatment of cavernous sinus dural arteriovenous fistula (CS-DAVF): case series and review of the literature |
| 2016 | Griauzde et al. | Dural carotid cavernous fistulas: endovascular treatment and assessment of the correlation between clinical symptoms and the Cognard classification system |
| 2015 | Thomas et al. | Proposal of Venous Drainage–Based Classification System for Carotid Cavernous Fistulae With Validity Assessment in a Multicenter Cohort |
| 2015 | Wen et al. | Transarterial Onyx Embolization for Patients with Cavernous Sinus Dural Arteriovenous Fistulas Who Have Failed Transvenous Embolization |
| 2015 | Hayashi et al. | Quadruple coaxial catheter system on transvenous embolization for dural arteriovenous fistula |
| 2014 | Kashiwazaki et al. | Delayed abducens nerve palsy after transvenous coil embolization for cavernous sinus dural arteriovenous fistulae |
| 2014 | Kiyosue et al. | Shunted pouches of cavernous sinus dural AVFs: evaluation by 3D rotational angiography |
| 2014 | Brunel et al. | Submandibular puncture of the facial vein: An original route for endovascular therapy of cavernous sinus dural fistulas |
| 2013 | Satow et al. | Superselective Shunt Occlusion for the Treatment of Cavernous Sinus Dural Arteriovenous Fistulae |
| 2013 | Kim et al. | Transvenous Embolization of Cavernous and Paracavernous Dural Arteriovenous Fistula through the Facial Vein: Report of 12 Cases |
| 2013 | Cha et al. | Clinical and angiographic results of patients with dural arteriovenous fistula |
| 2011 | Lekkhong et al. | Transvenous Embolization of Intracranial Dural Arteriovenous Shunts through Occluded Venous Segments: Experience in 51 Patients |
| 2010 | Zhang et al. | Transarterial and Transvenous Embolization for Cavernous Sinus Dural Arteriovenous Fistulae |
| 2010 | Li et al. | Transvenous embolization of cavernous sinus dural arteriovenous fistulas using detachable coils and Glubran 2 acrylic glue via the inferior petrosal sinus approach |
| 2010 | Chen et al. | Transvenous injection of n-Butyl cyanoacrylate combined with placement of coils in cavernous sinus for treatment of cavernous dural arteriovenous fistulae |
| 2010 | Bink et al. | Long-Term Outcome after Coil Embolization of Cavernous Sinus Arteriovenous Fistulas |
| 2010 | Cui et al. | Transvenous treatment of complex cavernous dural arteriovenous fistulae with Onyx and coils |
| 2008 | Jiang et al. | Transvenous Treatment of Cavernous Dural Arteriovenous Fistulae with Onyx and Coils |
| 2008 | Nishino et al. | Cranial nerve palsy following transvenous embolization for a cavernous sinus dural arteriovenous fistula: association with the volume and location of detachable coils |
| 2008 | Siqueira et al. | Endovascular Treatment of Carotid-Cavernous Fistulas: Review of 12 Cases |
| 2006 | Kim et al. | Results of Transvenous Embolization of Cavernous Dural Arteriovenous Fistula: A Single-Center Experience with Emphasis on Complications and Management |
| 2006 | Kirsch et al. | Endovascular management of dural carotid–cavernous sinus fistulas in 141 patients |
| 2006 | Takahashi et al. | Transvenous Embolization of Dural Arteriovenous Fistula of the Cavernous Sinus |
| 2004 | Satomi et al. | ANGIOGRAPHIC CHANGES IN VENOUS DRAINAGE OF CAVERNOUS SINUS DURAL ARTERIOVENOUS FISTULAE AFTER PALLIATIVE TRANSARTERIAL EMBOLIZATION OR OBSERVATIONAL MANAGEMENT: A PROPOSED STAGE CLASSIFICATION |
| 2004 | Tsumoto et al. | Analysis of Complications related to Endovascular Therapy for Dural Arteriovenous Fistulae |
| 2004 | Kiyosue et al. | Clinical use of a new mechanical detachable coil system for percutaneous intravenous embolization of cavernous sinus dural arteriovenous fistulas |
| 2003 | Klisch et al. | Transvenous treatment of carotid cavernous and dural arteriovenous fistulae: Results for 31 patients and review of the literature |
| 2003 | Hou et al. | Therapeutic embolization of cavernous sinus dural arteriovenous fistulas via transvenous approach |
| 2002 | Meyers et al. | Dural Carotid Cavernous Fistula: Definitive Endovascular Management and Long-term Follow-up |
| 2002 | Biondi et al. | Cavernous Sinus Dural Fistulae Treated by Transvenous Approach through the Facial Vein: Report of Seven Cases and Review of the Literature |
| 2001 | Liu et al. | Long-term clinical outcome of spontaneous carotid cavernous sinus fistulae supplied by dural branches of the internal carotid artery |
| 2000 | Kallmes et al. | Adjuvant use of epsilon-aminocaproic acid (Amicar) in the endovascular treatment of cranial arteriovenous fistulae |
| 2000 | Benndorf et al. | Superior ophthalmic vein approach for endovascular treatment of dural cavernous sinus fistulas |
| 1999 | Oishi et al. | Complications Associated with Transvenous Embolisation of Cavernous Dural Arteriovenous Fistula |
| 1999 | Liang et al. | Ophthalmologic outcome of transvenous embolization of spontaneous carotid-cavernous fistulas: a preliminary report |
| 1999 | Aihara et al. | Deterioration of Ocular Motor Dysfunction After Transvenous Embolization of Dural Arteriovenous Fistula Involving the Cavernous Sinus |
| 1999 | Iizuka et al. | Interventional Neuroradiology for Cavernous Dural Arterio-venous Fistula with Special Reference to Indications and Approach |
| 1998 | Veikko et al. | Endovascular treatment of carotidcavernous fistulae |
| 1997 | Roy et al. | The Role of Transvenous Embolization in the Treatment of Intracranial Dural Arteriovenous Fistulas |
| 1996 | Bavinzski et al. | Evolution of Different Therapeutic Strategies in the Treatment of Cranial Dural Arteriovenous Fistulas - Report of 30 Cases |
| 1995 | Miller et al. | Treatment of carotid-cavernous sinus fistulas using a superior ophthalmic vein approach |
| 1993 | Yamashita et al. | Transvenous embolization of dural caroticocavernous fistulae: technical considerations |
| 1989 | Halbach et al. | Transvenous Embolization of Dural Fistulas Involving the Cavernous Sinus |

**Supplemental Figure 1. Risk of bias evaluation**


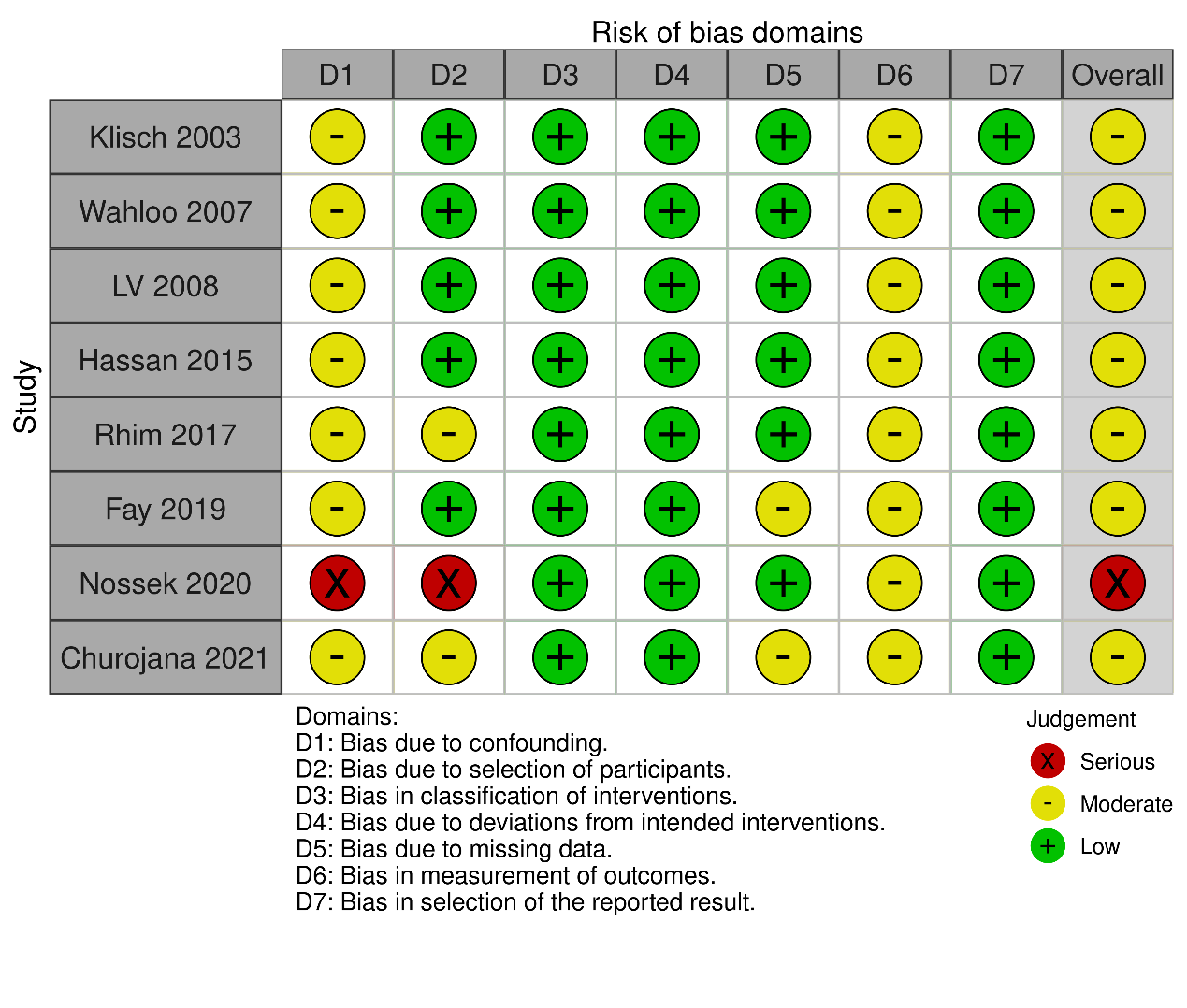

Supplement: Supplementary file 1 — Supplementary Information. [file 41598_2023_31864_MOESM1_ESM.docx]
